# FOXK2 regulates fatty acid metabolism and promotes cervical cancer progression by activating the mTOR/DRP1 signaling axis

**DOI:** 10.3389/fcell.2025.1615454

**Published:** 2025-06-26

**Authors:** Dan Liao, Saitian Zeng, Cuifen Li, Yuhong Yao, Min Guo, Yejia Cui, Haohai Huang

**Affiliations:** ^1^ Department of Gynaecology, The Affiliated Dongguan Songshan Lake Central Hospital, Guangdong Medical University, Dongguan, Guangdong, China; ^2^ Department of Clinical Laboratory, The Affiliated Dongguan Songshan Lake Central Hospital, Guangdong Medical University, Dongguan, Guangdong, China; ^3^ Clinical Translational Medical Center, The Affiliated Dongguan Songshan Lake Central Hospital, Guangdong Medical University, Dongguan, Guangdong, China

**Keywords:** cervical cancer, FOXK2, mTOR, DRP1, fatty acid metabolism

## Abstract

**Background:**

Cervical cancer is a prevalent malignancy among women, and its pathogenesis is highly complex. Lipid metabolism plays a crucial role in providing sufficient metabolites and energy for the rapid proliferation and progression of tumors, significantly influencing the advancement of cervical cancer. However, the specific lipid metabolism mechanisms remain to be thoroughly investigated. This study aims to elucidate the lipid metabolism mechanisms by which FOXK2 promotes the progression of cervical cancer.

**Methods:**

FOXK2 overexpression and knockdown cell lines were constructed, The cell activity and invasion were evaluated using CCK8, Edu, transwell, and flow cytometry. The oxygen consumption rate (OCR) values were detected by the XFe96 analyzer. The expression of fatty acid oxidation (FAO) related genes was analyzed by WB and qRT-PCR. The binding of FOXK2 to mTOR and mTOR to DRP1 was detected by co-immunoprecipitation (CoIP). Ultimately FOXK2-knockdown cells were applied to construct the Xenograft tumors in nude mice, and the relevant experiments were verified *in vivo*.

**Results:**

*In vitro* experiments, our findings demonstrated that FOXK2 enhances the proliferation and invasive capabilities of cervical cancer cells. FOXK2 expression was found to upregulate the expression of CPT1A, a key enzyme involved in FAO while downregulating the expression of critical lipogenic enzymes ACC1 and FASN. FOXK2 was shown to increase the phosphorylation levels of mTOR and interact with both mTOR and DRP1. Mechanistically, FOXK2 promotes lipid metabolic reprogramming in cervical cancer by interacting with the mTOR/DRP1 signaling axis. Furthermore, the role of FOXK2 in regulating lipid metabolism reprogramming in cervical cancer and its effects on the mTOR/DRP1 axis were validated in xenograft tumor models.

**Conclusion:**

FOXK2 interacts with and phosphorylates mTOR, which facilitates the expression of DRP1 and activates the mTOR/DRP1 signaling axis. This activation regulates lipid metabolic reprogramming and promotes the progression of cervical cancer.

## 1 Introduction

Cervical cancer is a prevalent form of cancer that primarily affects women. Unfortunately, patients diagnosed with advanced cervical cancer often face a grim prognosis (17%/5-year survival rate) (Bray et al., 2018). The treatments for cervical cancer involve surgical resection for early-stage cases and a combination of systemic chemotherapy and radiotherapy for advanced cases. Despite these treatment approaches, recurrence and metastasis remain significant challenges (Olusola et al., 2019). Consequently, there is a pressing need to delve into the underlying mechanisms of cervical cancer and develop novel therapeutic strategies.

Cancer cells exhibit distinct metabolic characteristics that provide them with a survival advantage over normal cells, enabling them to compete for the resources necessary for metabolic maintenance (Schiliro and Firestein, 2021). These traits propagate within tumor cells. Through metabolic reprogramming, cancer cells can synthesize large macromolecules and adenosine triphosphate (ATP), which are essential for sustaining cell growth, division, and survival (Ping et al., 2023). Recent studies indicate that fatty acid metabolism and related lipid metabolic pathways are closely associated with the malignant progression of cervical cancer (Yuan et al., 2024; Du et al., 2022; Zhong et al., 2024). Cancer-associated proteins can modulate lipid metabolism by activating crucial enzymes such as fatty acid synthase (FASN) and acetyl-CoA carboxylase (ACC), which are vital for *de novo* lipogenesis, promoting lipid accumulation and supplying energy for tumor growth (You et al., 2023). Fatty acid metabolism is regulated by both fatty acid synthesis and beta-oxidation processes. Fatty acids are first converted to fatty acyl-CoA, which is then transformed into fatty acylcarnitine. Carnitine palmitoyltransferase (CPT) transports fatty acylcarnitine into the mitochondria, where it is converted back into fatty acyl-CoA, subsequently undergoing beta-oxidation to yield acetyl-CoA (Chowdhury et al., 2023). Cancer cells could target various transcription factors and enzymes related to fatty acid metabolism through multiple mechanisms. This interaction encompasses several processes, including the synthesis, uptake, activation, oxidation, and transport of fatty acids (Gore et al., 2025). Furthermore, researchers are increasingly focused on how the breakdown of intracellular fat, transcription regulatory factors, alternative lipid metabolic pathways, and dietary factors influence the progression of cervical cancer.

Lipid metabolic processes typically occur in the mitochondria, however, the mechanisms that connect cellular metabolism with mitochondrial function remain unclear. Research conducted by Masahiro Morita and colleagues has identified the mTORC1/4E-BP/MTFP1/DRP1 signaling axis as a link between metabolism and mitochondria, regulating various aspects of tumor progression (Morita et al., 2017). The mTOR signaling pathway integrates extracellular signals and intracellular cues (such as growth factors, insulin, nutrients, and oxygen) to stimulate anabolic processes (such as protein and lipid synthesis) that promote cell growth and proliferation, while simultaneously inhibiting catabolic processes (Tewari et al., 2022).

Forkhead box K2 (FOXK2) has emerged as a significant promoter of cervical cancer (Liao et al., 2022), signaling its role as an oncogenic factor across various malignancies. This protein is implicated in several critical cellular processes, including cell proliferation, survival, and the response to DNA damage (Yu et al., 2024). Research has demonstrated a correlation between elevated FOXK2 expression and adverse clinical outcomes in patients with hepatocellular carcinoma (Zheng et al., 2023). Furthermore, heightened levels of FOXK2 have been documented in individuals with advanced rectal cancer (Zhang et al., 2021). Despite these findings, the precise downstream mechanisms through which FOXK2 influences the progression of cervical cancer remain inadequately characterized. Consequently, the objective of this study is to elucidate the molecular mechanisms underlying the role of FOXK2 in cervical cancer progression.

## 2 Materials and methods

### 2.1 Cell culture

HeLa (CL-0101) and SiHa (CL-0210) cells were obtained from Procell Life Science & Technology Co., Ltd. (Wuhan, China) and cultured with Dulbecco’s modified eagle medium (DMEM) supplemented with penicillin (100 U/mL), streptomycin (100 U/mL), and 10% fetal bovine serum (FBS) at 37°C and 5% CO_2_.

### 2.2 Reagents

Fatty acid oxidation promoter (FP) (ZLN-005, HY-17538, MCE, United States) and fatty acid oxidation inhibitor (FI) (Etomoxir, HY-50202, MCE, United States) were purchased from MCE. mTOR promoter (MP) (MHY1485, HY-B0795, MCE, United States) and mTOR inhibitor (MI) (Rapamycin, HY-10219, MCE, United States) were also purchased from MCE.

### 2.3 Cell transfection

Lentiviruses expressing sh-FOXK2, scrambled short hairpin RNA (shRNA), FOXK2, empty vector, and FOXK2 overexpression vector were purchased from Addgene (Beijing Zhongyuan Company). HeLa cells were used to establish stable FOXK2 knockdown models, and SiHa cells were used to establish stable FOXK2 overexpression models, based on the expression of FOXK2. A total of 5 × 10^4^ cells were plated into a 6-well plate and transfected with the indicated lentivirus or Lipofectamine 2000 (11668019, Invitrogen, United States) following the manufacturer’s instructions. Infected cells were selected using 2 μg/mL puromycin for ≥1 week, and the transfection efficiency was determined via quantitative reverse transcription polymerase chain reaction (qRT-PCR) analysis. The sequences of the FOXK2 shRNAs and pcDNAs used are detailed in Supplementary Table S1.

### 2.4 RT-PCR

The total RNA from cells was extracted using TRIzol (15596026CN, Invitrogen, United States), and the concentration of total RNA was measured. Complementary DNAs (cDNAs) were obtained by HiScript™ QRTSuperMix reverse-transcribed complementary DNA (R223-01, Vazyme, China). The primer sequences used are listed in Supplementary Table S2. GAPDH was used as the internal reference.

### 2.5 CCK8

A density of 5,000 cells was cultured in 96 well plates and incubated with lipopolysaccharide (LPS), 10% cell counting kit-8 (CCK8) (40203ES60, Yeasen, China) was added into the cells after 0, 1, 2, and 3 days treated. The absorbance was measured at 450 nm.

### 2.6 EdU assay

After treatment, the cells were stained with Edu reagent (10 μm; C0071S, Beyotime, China) at 37°C for 2 h. Fluorescence staining was performed using Azide 488 (green) and Hoechst 33342 (blue). The cell proliferation was calculated by Edu positive cells with Hoechst 33342 positive cells.

### 2.7 Transwell assay

Transplant cervical cancer cells were incubated in the top chamber with serum-free culture medium, while the lower involved 800 µL of complete substrate containing 10% FBS as a chemotactic agent. After 1 day of cultivation, the number of cells was stained with 0.5% crystal violet and calculated.

### 2.8 Cell apoptosis

The cells were harvested and stained with the Annexin V-fluorescein isothiocyanate (FITC)/propidium iodide (PI) apoptosis kit (AP107, MultiSciences, China). Then, CytoFLEX-3 flow cytometer (BD, United States) was used to identify cell apoptosis, and FlowJo software was applied to analyze the collected data.

### 2.9 Immunofluorescence

Cells were fixed with 4% paraformaldehyde at room temperature for 20 min. Then cells were blocked in 20% goat serum at room temperature for 1 h and incubated with the primary antibody at 4°C overnight. After washed with PBS 3 times, cells were then incubated with the secondary antibodies at room temperature for 1 h, followed by staining with Hoechst 33342. At last, cells were examined under a TCS-SP8 STED confocal laser scanning microscope (Leica, Frankfurt, Hesse-Darmstadt, Germany). The tissue samples of the xenograft were solidified in liquid nitrogen and were sent for immunofluorescent staining. The primary antibodies were as follows: CPT1A (1:500, 15184-1-AP, Proteintech), FASN (1:200, 10624-2-AP, Proteintech), ACC1 (1:200, 21923-1-AP, Proteintech).

### 2.10 OCR analysis

The extracellular flux analyzer XFe96 was used to analyze the FAO metabolism in HeLa and SiHa. 2.0 × 10^5^ cells were incubated with substrate-restricted medium and inoculated in FAO assay medium (KHB [111 mM NaCl, 4.7 mM KCl, 1.25 mM CaCl2, 2 mM MgSO4, 1.2 mM NaH2PO4], supplemented with 2.5 mM glucose, 0.5 mM L carnitine, and 5 mM HEPES). Bovine serum albumin (BSA) was immediately added to the well and the OCR was measured at 15 min. The experimental procedures were conducted according to the manufacturer’s instructions. The Agilent Seahorse XF Substrate Oxidation Stress Test Kit provides a method for investigating how cells oxidize three primary mitochondrial substrates: long-chain fatty acids, glucose or pyruvate, and glutamine. In this study, to explore the role of FOXK2 in lipid metabolism, palmitic acid was utilized as the substrate.

### 2.11 WB

The total protein was obtained using radioimmunoprecipitation assay (RIPA) buffer (Beyotime, P0013B, China). The total protein was separated by 10% SDS-PAGE (NCM, P2012, China), and then incubated with the primary antibody overnight at 4°C. The primary antibodies were as follows: p-mTOR (1:1000, AP0115, Abclonal), mTOR (1:1000, A2445, Abclonal), DRP1 (1:1000, A2586, Abclonal), CPT1A (1:1000, A5307, Abclonal), FASN (1:1000, A0461, ABclonal), ACC1 (1:1000, A15606, ABclonal), and GAPDH (1:50000, A19056, ABclonal). Subsequently, incubated with the secondary antibody (1:5000, Goat Anti-Rabbit IgG, Invitrogen, 31430/31460, United States) for 30 min. Finally, the protein blots were analyzed by the Alliance Q9 Advanced (UVITEC, UK).

### 2.12 CoIP

50 μL of A/G protein beads were inhaled into 400 μL of IP buffer and rinsed twice. The amount of primary antibody was added in 400 μL IP buffer and incubated at 4°C for 2 h. Finally, precipitate the beads and perform the Western blot (WB) analysis on the supernatant. To validate the binding site between mTOR and FOXK2, DeepProSite (https://inner.wei-group.net/DeepProSite/) was used to predict the specific binding site of mTOR and FOXK2. A mutant plasmid for this site was then constructed (GenePharma, China). HeLa cells were transfected with the flag-FOXK2 -R mutant and the IP experiment was performed using an endogenous mTOR antibody. After a WB experiment, we incubated with a flag primary antibody to determine whether it could bind to flag-FOXK2-R. We then transfected cells with the myc-mTOR-R mutant and performed an IP experiment using an endogenous FOXK2 antibody. After a WB experiment, we incubated with a myc primary antibody to see if it could bind to myc-mTOR-R.

### 2.13 Xenograft tumors

Male nude BALB/c mice (4–6 weeks, weight 18–22 g) were placed in a 12/12 h light/dark cycle at 20–24°C and 50%–60% humidity, with free food and water. HeLa cells (4.0 × 10^7^ cells/mL) transfected with an empty vector or a FOXK2 shRNA vector, and SiHa cells (4.0 × 10^7^ cells/mL) transfected with an empty vector or a FOXK2 overexpression vector were suspended in phosphate buffer saline (PBS) and injected subcutaneously in the back neck of nude mice. The mice were randomly divided into Control, sh-FOXK2, sh-FOXK2 + MP groups, and Control, OE-FOXK2, and OE-FOXK2 + MI groups. In addition, during the treatment of mice with MP and MI, a concentration of 10 mg/kg of MP and 2 mg/kg of MI were administered via intraperitoneal injection (once every other day). On Days 7, 14, 21, 28, and 35 following injection, the tumor size was estimated from luciferase volume measurements. Mice were anesthetized with 2% pentobarbital sodium at a dose of 50 mg/kg on day 35 and euthanized by cervical dislocation. Subsequently, tumor tissues from all mice were collected and weighed. This study was approved by the Institutional Animal Care and Use Committee of the Hubei Provincial Center for Disease Control and Prevention.

### 2.14 H&E staining

The tumor tissue was fixed in 10% paraformaldehyde. The tissue was longitudinally embedded in paraffin with a slice thickness of 3–5 μm and then stained with hematoxylin and eosin (H&E). The glass slide using an orthogonal microscope (Nikon Eclipse E100; Nikon, Tokyo, Japan) was observed and images were captured using an imaging system (Nikon DS-U3; Nikon, Tokyo, Japan).

### 2.15 Immunohistochemistry (IHC)

Tissue samples from subcutaneous tumors of cervical cancer were processed. These samples underwent deparaffinization, rehydration, antigen retrieval, and peroxidase blocking before incubation with Ki-67 antibody (1:4000, 27309-1-AP, Proteintech) to assess cell proliferation. Staining was carried out using secondary antibodies and DAB substrate, followed by counterstaining with hematoxylin. The stained sections were observed under a microscope.

### 2.16 Statistic analysis

The SPSS 20.0 software (IBM, Chicago, Illinois, United States) was used to analyze the data. Numerical data was displayed as mean +standard deviation. An Independent sample t-test was used to compare the differences between the two groups. One-way ANOVA analysis was performed for comparisons involving three or more groups, and ImageJ software was utilized to analyze the immune fluorescence in 3D images. P < 0.05 was considered statistically significant. All experiments were repeated at least three times to ensure reproducibility.

## 3 Results

### 3.1 FOXK2 promotes the progression of cervical cancer

**FIGURE 1:**
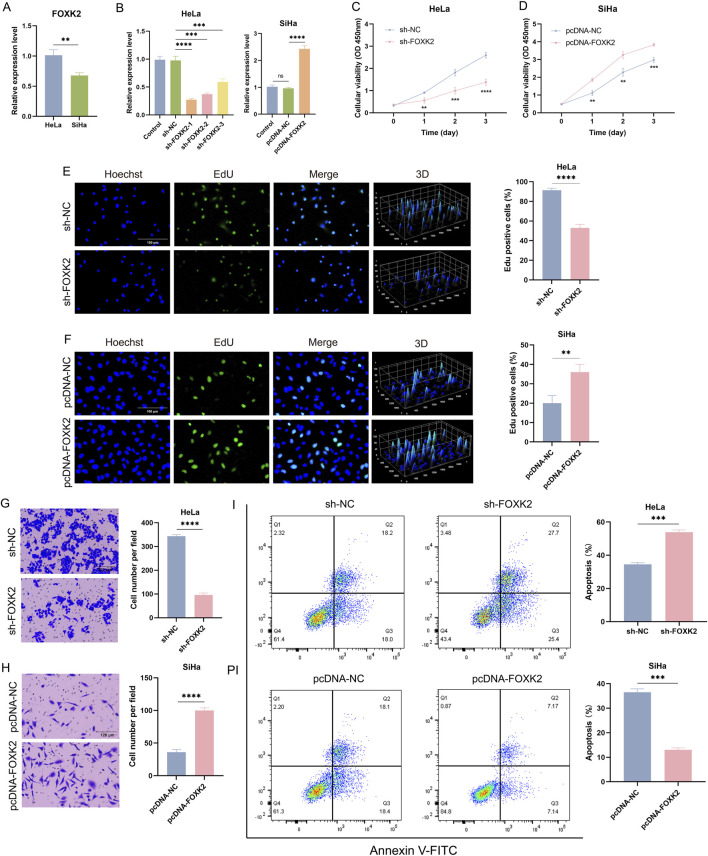
FOXK2 promotes the progression of cervical cancer. **(A)** The mRNA level of FOXK2 in HeLa and SiHa. **(B)** Constructing sh-FOXK2 in HeLa cells and pcDNA-FOXK2 in SiHa cells. **(C)** Cellular activity of HeLa cells after sh-FOXK2. **(D)** Cellular activity of SiHa cells after pcDNA-FOXK2. **(E)** The Edu level of HeLa cells after sh-FOXK2. **(F)** The Edu level of SiHa cells after pcDNA-FOXK2. **(G)** The invasion of HeLa cells after sh-FOXK2. **(H)** The invasion of SiHa cells after pcDNA-FOXK2. **(I)** The apoptosis of HeLa cells after sh-FOXK2 and SiHa cells after pcDNA-FOXK2. **p 0.01, ***p 0.001, ****p 0.0001, t-test based p-value.

Firstly, qRT-PCR experiments were conducted in HeLa and SiHa cervical cancer cell lines. The results demonstrated that FOXK2 is highly expressed in the HeLa cervical cancer cell line, whereas it is expressed at a lower level in SiHa cells (Figure 1A). Based on these findings, to confirm the role of FOXK2 in the progression of cervical cancer. We constructed stable FOXK2 knockdown cell lines in HeLa cells using a short hairpin RNA (shRNA) strategy and established FOXK2 overexpression cell lines in SiHa cells using a plasmid (Figure 1B). By comparing the knockdown efficiencies of various shRNA constructs, we found that shFOXK2-1 exhibited the highest efficiency. Therefore, we utilized shFOXK2-1 for further experiments aimed at investigating its effects on cervical cancer cells. The effects of FOXK2 on cellular proliferation were assessed using CCK-8 and EdU proliferation assays, which demonstrated that FOXK2 significantly enhances the proliferative capacity of cervical cancer cells (Figures 1C–F). Furthermore, Transwell assays revealed that FOXK2 markedly promotes the migratory capability of cervical cancer cells (Figures 1G,H). Finally, flow cytometry analysis indicated that FOXK2 knockdown increased the number of apoptotic cells, while overexpression of FOXK2 significantly reduced apoptosis (Figure 1I). These results collectively suggest that FOXK2 facilitates the proliferation and invasion of cervical cancer cells while concurrently inhibiting apoptosis.

### 3.2 FOXK2 promotes fatty acid metabolism

**FIGURE 2:**
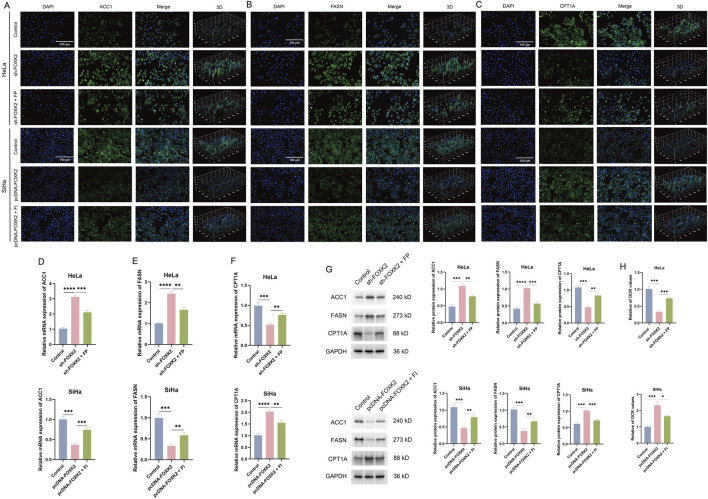
FOXK2 promotes fatty acid metabolism in cervical cancer cells. **(A–C)** The expression of ACC1, FASN, and CPT1A was detected with immunofluorescence in cervical cancer cells. **(D–F)** The expression of ACC1, FASN, and CPT1A was detected with qRT-PCR in cervical cancer cells. **(G)** The protein expression of ACC1, FASN, and CPT1A was detected with WB in cervical cancer cells. **(H)** The OCR level in cervical cancer cells. *p 0.05, **p 0.01, ***p 0.001, ****p 0.0001, t-test based p-value.

To investigate the regulatory role of FOXK2 in lipid metabolism, we conducted research focusing on two key aspects: FAO and fatty acid synthesis. CPT1A serves as the rate-limiting enzyme in fatty acid oxidation, while FASN and ACC1 are crucial enzymes involved in fatty acid synthesis. In cervical cancer cells, we utilized immunofluorescence experiments and discovered that the knockdown of FOXK2 led to a reduction in the expression level of CPT1A, whereas the expression levels of FASN and ACC1 increased. In contrast, overexpression of FOXK2 produced the opposite effect. Given the significant coordinated changes observed between FOXK2 and CPT1A, we treated the cells with the FP and FI. The immunofluorescence results indicated that upon knockdown of FOXK2 and subsequent treatment with FP, there was an increase in CPT1A and a decrease in FASN and ACC1 expression compared to the group treated with sh-FOXK2 alone. Conversely, in the scenario where FOXK2 was overexpressed and cells were treated with FI, there was a downregulation of CPT1A and an upregulation of FASN and ACC1 expression relative to the pcDNA-FOXK2 group. These findings suggest that FOXK2 can regulate lipid metabolism by promoting FAO while simultaneously inhibiting fatty acid synthesis (Figures 2A–C). The results were corroborated by subsequent WB and qRT-PCR experiments (Figures 2D–G). Additionally, we characterized the FAO rate using palmitic acid as a substrate. Utilizing the Seahorse XF technology to measure the oxygen consumption rate (OCR), we found that knockdown of FOXK2 significantly decreased the OCR value after 15 min, whereas overexpression of FOXK2 resulted in a notable increase in OCR. Treatment with FP and FI reversed these trends (Figure 2H). These results indicated that overexpression of FOXK2 significantly enhances the FAO process.

### 3.3 FOXK2 regulates the mTOR/DRP1 signaling axis

**FIGURE 3:**
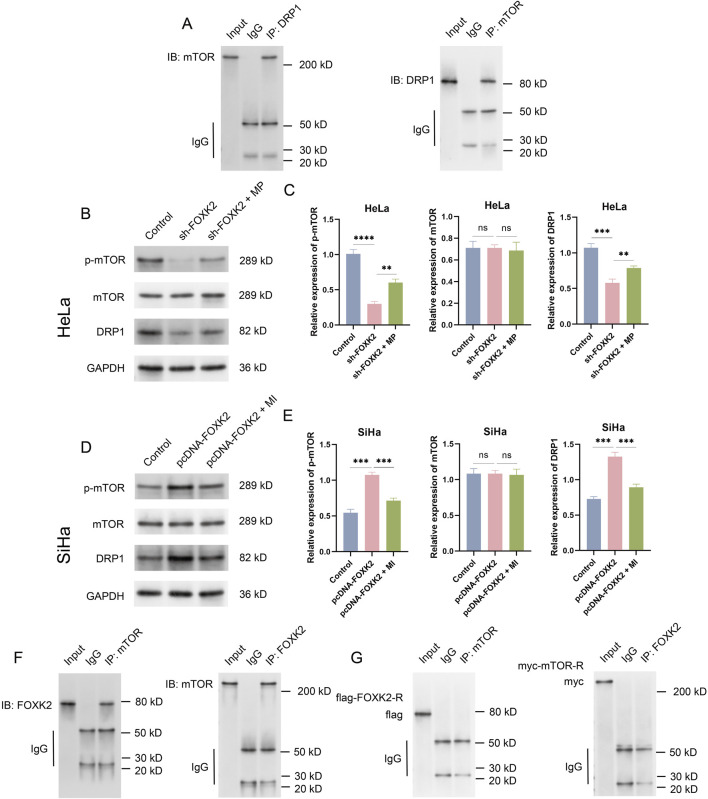
FOXK2 upregulates the mTOR/DRP1 signaling axis. **(A)** Immunoprecipitation verification of the binding of mTOR and DRP1 in HeLa cells. **(B)** The protein level of p-mTOR, mTOR, and DRP1 in HeLa cells. **(C)** The Protein grayscale analysis in HeLa cells. **(D)** The protein level of p-mTOR, mTOR, and DRP1 in SiHa cells. **(E)** The Protein grayscale analysis in SiHa cells. **(F)** Immunoprecipitation verification of the binding of mTOR and FOXK2 HeLa cells. **(G)** Immunoprecipitation verification of the binding of mutant FOXK2 to mTOR and mutant mTOR to FOXK2 in HeLa cells. **p 0.01, ***p 0.001, ****p 0.0001, t-test based p-value.

mTOR and DRP1 have been proven to play crucial roles in the regulation of FAO metabolism within key signaling pathways. To elucidate the relationship between mTOR and DRP1, we initially conducted Co-IP experiments, which revealed a significant interaction between mTOR and DRP1 (Figure 3A). Following this, we aimed to investigate the connection between FOXK2 and the mTOR/DRP1 signaling axis. Using WB and qRT-PCR experiments in HeLa and SiHa cell lines, we found that knockdown of FOXK2 resulted in a significant decrease in the phosphorylation levels of the downstream protein mTOR and the expression of DRP1, while the overall expression of mTOR remained unchanged. The introduction of an mTOR agonist was able to reverse these trends, whereas overexpression of FOXK2 yielded the opposite results. Additionally, treatment with an MI also reversed these effects (Figures 3B–E). Moreover, our Co-IP experiments demonstrated that FOXK2 could directly interact with mTOR (Figures 3F,G). Therefore, it can be concluded that FOXK2 upregulates the mTOR/DRP1 signaling axis, facilitating its corresponding biological functions.

### 3.4 FOXK2 promotes fatty acid metabolism through mTOR

**FIGURE 4:**
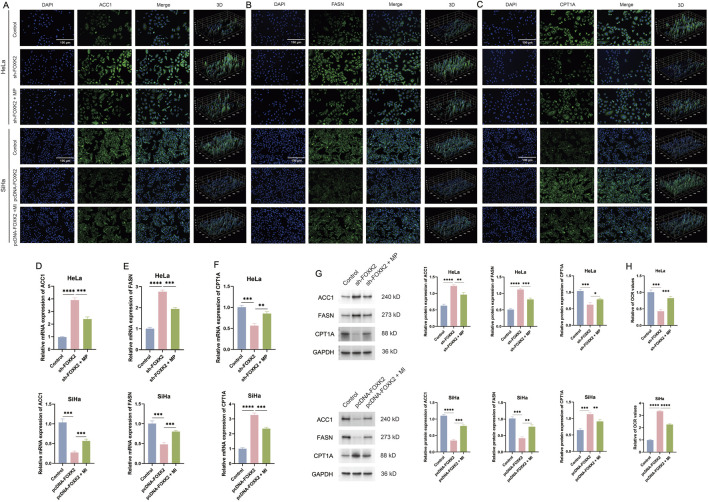
FOXK2 promotes fatty acid metabolism through mTOR. **(A–C)** The immunofluorescence results of ACC1, FASN, and CPT1A in cervical cancer cells. **(D–F)** The qRT-PCR results of ACC1, FASN, and CPT1A in cervical cancer cells. **(G)** The WB results of ACC1, FASN, and CPT1A in cervical cancer cells. **(H)** The OCR results of cervical cancer cells. *p 0.05, **p 0.01, ***p 0.001, ****p 0.0001, t-test based p-value.

Given that both FOXK2 and mTOR facilitate lipid metabolic synthesis while inhibiting lipid degradation, we aimed to investigate the relationship between the mTOR/DRP1 signaling axis and lipid metabolism. To this end, we conducted experiments involving the knockout of FOXK2 followed by treatment with MP, as well as the overexpression of FOXK2 in conjunction with MI treatment. Immunofluorescence assays revealed that silencing FOXK2 expression before MP treatment resulted in decreased expression levels of ACC1 and FASN, along with an increase in CPT1A expression compared to the sh-FOXK2 group. In contrast, overexpression of FOXK2 combined with MI treatment led to a significant upregulation of ACC1 and FASN, while simultaneously downregulating CPT1A compared to the pcDNA-FOXK2 group (Figures 4A–C). Subsequent WB and qRT-PCR analyses also corroborated these findings (Figures 4D–G). These results suggest that the regulatory effect of FOXK2 on lipid metabolism is modulated by the mTOR signaling pathway. Additionally, during the proliferation of tumor cells, we observed a significant increase in the OCR after 15 min of MP treatment following the knockout of FOXK2 compared to the sh-FOXK2 group. In contrast, the OCR in the group overexpressing FOXK2 in conjunction with MI treatment was notably lower compared to the pcDNA-FOXK2 group (Figure 4H). Based on these observations, we propose that FOXK2 may promote lipid metabolic reprogramming through the regulation of the mTOR/DRP1 signaling axis. This highlights the potential interplay between FOXK2 and mTOR in orchestrating lipid metabolism, with implications for understanding metabolic reprogramming in malignancies.

### 3.5 FOXK2 induced tumor progression via mTOR/DRP1 signaling axis *in vivo*

**FIGURE 5:**
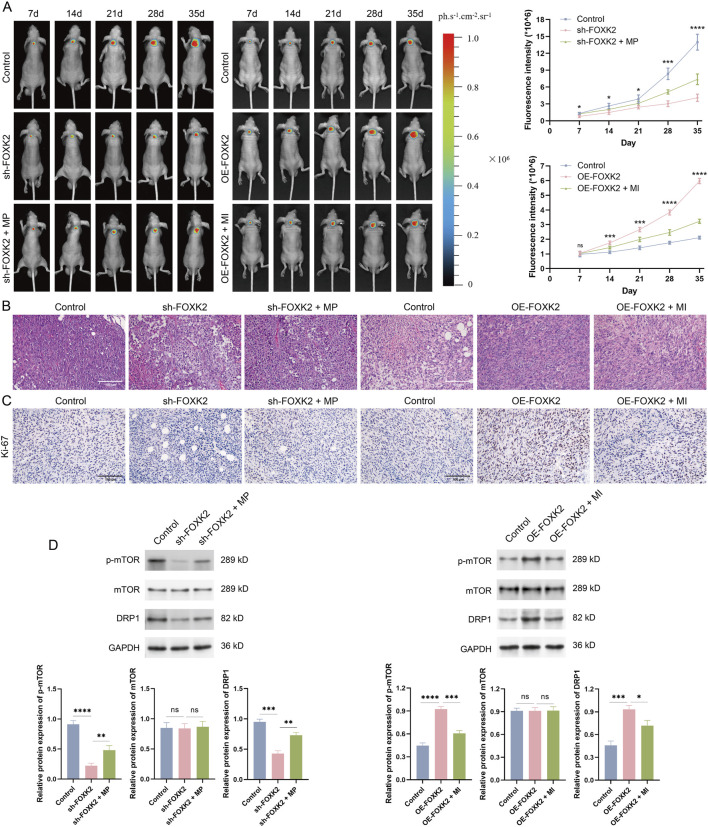
FOXK2 induces tumor progression via mTOR/DRP1 signaling axis *in vivo*. **(A)** Representative tumor bioluminescence images and the bioluminescence intensity of mice at 7, 14, 21, 28, and 35 days after tumor implantation in a xenograft model. **(B)** HE staining results of tumor tissues to evaluate tumor infiltration. **(C)** IHC analysis was conducted to evaluate the *in vivo* proliferative capacity of tumors. **(D)** The protein level of p-mTOR, mTOR, and DRP1 in tumor tissues. *p 0.05, **p 0.01, ***p 0.001, ****p 0.0001, t-test based p-value.

We first established a cervical cancer mouse model and created experimental groups, including the FOXK2 knockout combined with the MP treatment group, and the FOXK2 overexpression combined with the MI treatment group. Tumor formation in the mice was monitored during weeks 1 through 5. Fluorescence results indicated that isolated knockout of the FOXK2 gene significantly inhibited the proliferation of cervical cancer cells, with the highest inhibition rate observed in week 5. Moreover, there was a notable reduction in the infiltration and invasion of tumor cells. However, when FOXK2 was knocked down and MP treatment was administered, tumor growth markedly accelerated, and the degree of infiltration increased. Similar conclusions were drawn from the FOXK2 overexpression group and the MI treatment group (Figure 5A). Histological analysis through HE staining of different treatment groups revealed that the cells in the FOXK2 knockout group exhibited more intact morphology, with a more orderly arrangement and a lower degree of malignancy. In contrast, overexpression of FOXK2 significantly exacerbated tumor malignancy and conferred a more aggressive cellular phenotype. Furthermore, MP treatment promoted tumor progression, reversing the inhibitory effect of FOXK2 silencing on tumor proliferation, while MI treatment markedly weakened the malignant characteristics associated with FOXK2 overexpression (Figure 5B). IHC analysis demonstrated that FOXK2 promoted the expression of Ki-67, with both MP and MI treatments capable of reversing the effects of FOXK2 knockout and overexpression on Ki-67 expression, respectively (Figure 5C). Additionally, results from WB and qRT-PCR experiments indicated that FOXK2 knockout led to decreased phosphorylation of mTOR and reduced DRP1 expression levels, with similar conclusions observed in the FOXK2 overexpression group and the MI treatment group (Figure 5D). These findings suggest that FOXK2 may mediate tumor progression *in vivo* through the mTOR/DRP1 signaling axis, highlighting its potential role in influencing tumor behavior and therapeutic response in cervical cancer. Further exploration of this regulatory mechanism could offer valuable insights for developing targeted therapies aimed at metabolic pathways in cancer.

### 3.6 FOXK2 enhances fatty acid metabolism *in vivo*

**FIGURE 6:**
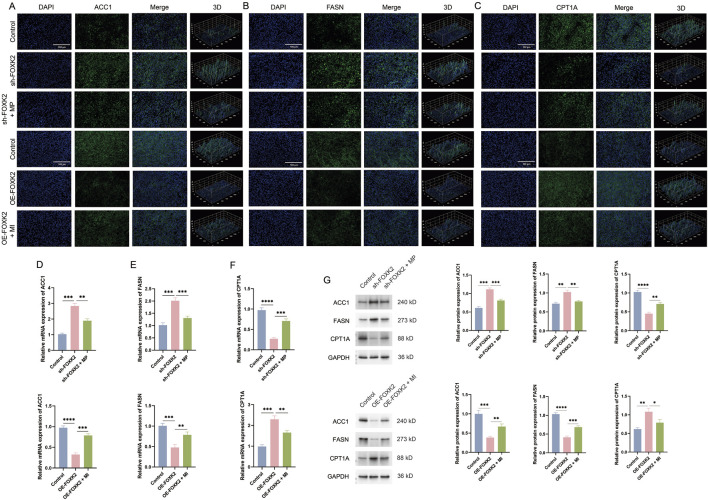
FOXK2 enhances fatty acid metabolism *in vivo*. **(A–C)** The results of the immunofluorescence assays demonstrated changes in the expression levels of ACC1, FASN, and CPT1A in tumor tissues. **(D–F)** The results of the qRT-PCR experiments indicated significant changes in the mRNA expression levels of ACC1, FASN, and CPT1A in tumor tissues. **(G)** The results of the WB experiments revealed significant changes in the protein expression levels of ACC1, FASN, and CPT1A in tumor tissues. *p 0.05, **p 0.01, ***p 0.001, ****p 0.0001, t-test based p-value.

To further investigate the role of FOXK2 in regulating lipid metabolism in a cervical cancer mouse model, we assessed the expression levels of ACC1, FASN, and CPT1A in tumor tissues. Immunofluorescence staining results indicated that the isolated knockout of FOXK2 resulted in a significant increase in the expression of ACC1 and FASN, while the expression levels of the key metabolic gene CPT1A were decreased. This finding is consistent with results obtained at the cellular level. However, following treatment with MP, there was a reduction in the expression levels of ACC1 and FASN, while CPT1A expression increased. Conversely, overexpression of FOXK2 led to a decrease in ACC1 and FASN expression, accompanied by an increase in CPT1A levels. After treatment with MI, ACC1 and FASN expression increased, while CPT1A expression levels decreased (Figures 6A–C). We further validated these findings through WB and qRT-PCR analyses, which confirmed the expression levels of ACC1, FASN, and CPT1A following MP and MI treatments (Figures 6D–G). The results were consistent with those obtained from immunofluorescence assays, reinforcing the conclusion that FOXK2 can influence lipid metabolism in cervical cancer. These findings suggest that FOXK2 may promote metabolic reprogramming of lipids *in vivo*, highlighting its potential role in modulating lipid metabolic pathways in the context of cervical cancer. Further elucidation of the mechanisms involved could provide insights into therapeutic strategies targeting metabolic reprogramming in malignancies.

## 4 Discussion

Cervical cancer is the fourth most commonly diagnosed malignant tumor among women worldwide, with approximately 660,000 new cases reported in 2022. According to the World Health Organization (WHO) global strategy for the elimination of cervical cancer, which aims for an incidence rate of less than 4 cases per 100,000 women annually (Xu et al., 2025), it is estimated that around 350,000 women globally (Sung et al., 2021), including 44,750 women in China (Qi et al., 2023), could potentially avoid death from this disease. Despite a significant decline in cervical cancer incidence over the past 3 decades, it remains a pressing global public health concern. Given the clearly defined etiology, early and accurate screening for high-risk populations is crucial in the fight against this cancer. In addition to current screening technologies, ongoing research is exploring more precise and efficient screening methods, such as HPV integration screening, epigenetic markers, and liquid biopsies. For advanced cervical cancer, new therapies bring hope in addressing treatment challenges, with combination therapies aiming to overcome these barriers. However, the effectiveness of treatments varies significantly, and the management of cervical cancer will increasingly rely on interdisciplinary collaboration that integrates molecular biology, immunology, pharmacology, and precision engineering (Xu et al., 2025). This integrated approach aims to develop safer, more effective, and personalized treatment strategies to enhance patient survival rates and quality of life. Therefore, a thorough investigation into the mechanisms of cervical cancer pathogenesis could provide new therapeutic targets for the treatment of this disease.

In this study, we identified the critical role of FOXK2 in the progression of cervical cancer. FOXK2 is a member of the FOXK family of transcription factors, which regulate various cellular processes, including metabolism, cell cycle progression, proliferation, survival, differentiation, and apoptosis (Sakaguchi et al., 2019; van der Heide et al., 2015; Ji et al., 2021; Zhang et al., 2018). Our findings indicate that FOXK2 can modulate lipid metabolic reprogramming, thereby promoting the onset and progression of cervical cancer. Furthermore, we confirmed that enhanced lipid metabolic reprogramming indeed facilitates tumor advancement. Cellular metabolism plays a vital role in meeting energy demands and providing essential substrates necessary for cellular growth and function. Lipid metabolism primarily occurs in mitochondria or peroxisomes and involves processes such as fatty acid synthesis, fatty acid esterification, acyl-CoA transfer, and the β-oxidation of coenzyme A. Previous studies have demonstrated that lipid metabolism can promote tumor progression. For instance, adipocytes have been found to facilitate cancer metastasis and invasion by reprogramming fatty acid metabolism (Cai et al., 2023). FAO metabolizes and breaks down fatty acids through a series of cyclic reactions within the mitochondria, converting long-chain fatty acids into acetyl-CoA and generating substantial ATP and reducing equivalents, which serve as primary energy sources for certain malignant tumor cells (Chen et al., 2022). Recent research has emphasized the “lipolytic phenotype” observed in cancer (Ma et al., 2018), indicating the presence of abnormal fatty acid catabolism in various tumor tissues (Quan et al., 2022). Targeting lipid metabolism has emerged as a promising new strategy for cancer treatment. Moreover, lipid metabolic reprogramming is closely associated with the initiation and progression of cervical cancer. FAO-derived acetyl-CoA enhances the acetylation of histone H3 Lysine 27 in stemness gene promoters, increasing stemness and lymphatic metastasis in lipid-rich microenvironments. The genetic and pharmacological inhibition of CPT1A function significantly suppressed the metastatic colonization of CCa cells in tumor-draining lymph nodes (Yuan et al., 2024). FASN regulates cholesterol reprogramming, subsequently activating the lipid raft-related c-Src/AKT/FAK signaling pathway, which results in enhanced migration and invasion of cervical cancer cells. Conversely, FASN promotes lymphangiogenesis and metastasis in cervical cancer via the secretion of PDGF-AA/IGFBP3 (Du et al., 2022). FAO supports lymph node metastasis in cervical cancer through acetyl-CoA-mediated stress responses. In addition, numerous other genes can promote the progression of cervical cancer by modulating lipid metabolic reprogramming (Zhong et al., 2024; Zhang et al., 2020; Han et al., 2024). Additionally, several studies have indicated that lipid metabolic reprogramming represents a potential therapeutic target for cervical cancer (Lang et al., 2022; Zeng et al., 2022).

The process of fatty acid synthesis is frequently upregulated in cancer, with both fatty acid synthesis and uptake stimulated in response to mTOR signaling. mTORC1 activates numerous metabolic pathways, including oxidative phosphorylation through the enhancement of mitochondrial biogenesis, facilitating *de novo* nucleotide synthesis, and promoting lipogenesis (Koundouros and Poulogiannis, 2020). Both mTORC1 and mTORC2 contribute to the upregulation of SREBP1, which acts as a key transcription factor driving the expression of fatty acid synthases. This cascade subsequently leads to the increased expression of enzymes involved in fatty acid synthesis, including ACLY, ACC1, FASN, and SCD1, while simultaneously downregulating critical enzymes responsible for fatty acid oxidation, such as CPT1A (Mossmann et al., 2018). Targeting mTOR signaling as a regulatory mechanism that promotes fatty acid uptake and synthesis may offer a strategic approach to overcoming potential resistance mechanisms in cancer treatment. Moreover, mitochondria serve as crucial dynamic metabolic hubs, with their structural integrity being pivotal for the regulation of lipid metabolism. These organelles continually undergo cycles of fusion and fission, allowing cells to adapt to both intracellular and extracellular signals (Youle and van der Bliek, 2012). Cancer cells exploit these adaptive mitochondrial dynamics to fulfill energy demands, control reactive oxygen species levels, reprogram cellular metabolism, and survive under conditions of environmental or nutritional stress. Mitochondrial plasticity supports FAO and metabolic reprogramming, enabling a response to cellular stress, nutritional availability, and energy requirements, thereby enhancing the survival of disseminated tumor cells (Molina et al., 2009; Rambold et al., 2011). DRP1 is a key protein regulating mitochondrial fission, typically distributed freely in the cytoplasm, and can promote tumor progression through various pathways (Parida et al., 2023; Ding et al., 2024; Li et al., 2022). Research has shown that silencing Drp1 eliminates SB-induced G2/M cell cycle arrest in cervical cancer cells by inhibiting mitochondrial fission pathways (You et al., 2020). Numerous genes that modulate DRP1 expression can alter mitochondrial structure or function, thereby facilitating the development of cervical cancer (Jin et al., 2018; Xu et al., 2023; Bhushan et al., 2009; Khan et al., 2011). Research has indicated that the depletion of DRP1, which is enriched in latency-associated cells and plays a role in constraining mitochondrial plasticity, results in increased accumulation of lipid droplets, impaired fatty acid oxidation, and diminished metastatic potential. Additionally, pharmacological inhibition of DRP1 using small-molecule inhibitors that penetrate the central nervous system has been shown to reduce metastatic burden (Parida et al., 2023). Xiong et al. demonstrated that silencing DRP1 disrupts cellular metabolism and prevents fatty acid-induced metabolic reprogramming by inhibiting fatty acid utilization. Functionally, DRP1 knockout attenuates Wnt/β-catenin signaling by obstructing the fatty acid oxidation-dependent acetylation of β-catenin (Xiong et al., 2022). Furthermore, it has been reported that DRP1-mediated mitochondrial fission is crucial for maintaining the balance between fatty acid storage and utilization by promoting mitochondrial uptake of fatty acids (Song et al., 2021). Overall, the mTOR-DRP1 signaling axis plays a significant role in regulating various aspects of metabolism, aging, cell survival, and autophagy (Morita et al., 2017; Yu et al., 2020; Meyer et al., 2024).

Previous literature has demonstrated that FOXK2 can regulate the phosphorylation of mTOR. Research on FOXK2’s regulation of tumor progression via metabolic pathways has primarily focused on glycolytic pathways. Studies have shown that both FOXK1 and FOXK2 enhance aerobic glycolysis by upregulating key enzymes, including hexokinase-2, phosphofructokinase, pyruvate kinase, and lactate dehydrogenase. Simultaneously, they inhibit the further oxidation of pyruvate in mitochondria by increasing the activity of pyruvate dehydrogenase kinases 1 and 4 (PDK1 and PDK4) (Sukonina et al., 2019). Notably, PDK2 has been reported to directly bind to the forkhead-associated domain of FOXK2, promoting the phosphorylation of FOXK2 at Thr13 and Ser30, which enhances its transcriptional activity. Additionally, FOXK2 transcriptionally regulates the expression of PDK2, thereby creating a positive feedback loop that sustains glycolysis in ovarian cancer cells (Zhang et al., 2024). Furthermore, FOXK1 and FOXK2 are downstream targets of insulin signaling. Upon insulin stimulation, these proteins translocate from the cytoplasm to the nucleus, a process mediated by the Akt-mTOR pathway. Conversely, their retention in the cytoplasm under basal conditions is dependent on the activity of glycogen synthase kinase 3. Silencing FOXK1 and FOXK2 in hepatocytes leads to the upregulation of apoptosis-related genes and the downregulation of genes involved in the cell cycle and lipid metabolism. This shift is associated with reduced cell proliferation and alterations in mitochondrial fatty acid metabolism (Sakaguchi et al., 2019). In summary, FOXK1 and FOXK2 play critical roles in interacting with FOXO1 following insulin stimulation, significantly influencing apoptosis, metabolism, and mitochondrial function. These findings underscore the importance of FOXK1 and FOXK2 in the metabolic reprogramming that supports tumor progression and suggests promising avenues for therapeutic intervention in cancer treatment.

However, this study is not without limitations. Firstly, we have not comprehensively validated the correlation between FOXK2 and lipid metabolism through more refined mechanistic studies. We did not assess the expression of FOXK2 in human tissue samples, nor did we thoroughly investigate the upstream mechanisms regulating FOXK2. Our verification was limited to the expression levels of key enzymes. Secondly, our exploration of the mechanisms is somewhat superficial, and a tighter connection should be established between the mTOR/DRP1 axis, FOXK2, and lipid metabolic reprogramming. Additionally, our validation at the cellular level is somewhat insufficient. Lastly, a more multifaceted experimental design is necessary, as our current approach was somewhat singular in focus.

In summary, the overexpression of FOXK2 can enhance FAO metabolism in tumor cells through mechanisms that can be outlined as follows: it increases the binding affinity of FOXK2 to the mTOR, upregulates the expression of DRP1, and promotes FAO, thereby driving tumor progression. Future research should delve deeper into identifying which specific binding sites on FOXK2 contribute to the phosphorylation of mTOR. The findings of this study suggest that FOXK2, along with its associated genes and pathways related to FAO, could serve as promising new targets for the development of novel immunotherapeutics for the treatment of cervical cancer.

## Data Availability

The original contributions presented in the study are included in the article/Supplementary Material, further inquiries can be directed to the corresponding authors.

## Abbreviations

OCR, Oxygen consumption rate; FAO, Fatty acid oxidation; CoIP, Co-immunoprecipitation; ATP, Adenosine triphosphate; FASN, Fatty acid synthase; ACC, Acetyl-CoA carboxylase; CPT, Carnitine palmitoyltransferase; FOXK2, Forkhead box K2; DMEM, Dulbecco’s modified eagle medium; FBS, Fetal bovine serum; shRNA, Short hairpin RNA; qRT-PCR, Quantitative reverse transcription polymerase chain reaction; cDNA, Complementary DNA; LPS, Lipopolysaccharide; CCK8, Cell counting kit-8; FITC, Fluorescein isothiocyanate; PI, Propidium iodide; BSA, Bovine serum albumin; RIPA, Radioimmunoprecipitation assay; PBS, Phosphate buffer saline; MP, mTOR promoter; H&E, Hematoxylin and eosin; IHC, Immunohistochemistry; shRNA, Short hairpin RNA; OCR, Oxygen consumption rate; WHO, World Health Organization; FP, FAO promoter; FI, FAO inhibitor; MI, mTOR inhibitor; Acyl-CoA, Acyl-coenzyme A; GTPase, Guanosine triphosphatase; ROS, Reactive oxygen species; ATP, Adenosine triphosphate.
