# Supplementary material for: FOXK2 regulates fatty acid metabolism and promotes cervical cancer progression by activating the mTOR/DRP1 signaling axis
Source: Front Cell Dev Biol. 2025 Jun 26;13:1615454. doi: 10.3389/fcell.2025.1615454 (PMC12240978; doi:10.3389/fcell.2025.1615454)
Supplement: Supplementary file 1 [file Table1.docx]

**Table S1.** The sequences for FOXK2 shRNA and pcDNA.

| Name | sense 5’-3’ | antisense 5’-3’ |
| --- | --- | --- |
| sh-FOXK2-1 | CCGGCCAGCCTCTGAAAGCAAATTACTCGAGTAATTTGCTTTCAGAGGCTGGTTTTT | AATTAAAAACCAGCCTCTGAAAGCAAATTACTCGAGTAATTTGCTTTCAGAGGCTGG |
| sh-FOXK2-2 | CCGGCGAGTTCGAGTATCTGATGAACTCGAGTTCATCAGATACTCGAACTCGTTTTT | AATTAAAAACGAGTTCGAGTATCTGATGAACTCGAGTTCATCAGATACTCGAACTCG |
| sh-FOXK2-3 | CCGGCCCGAGCACAAACATCAAGATCTCGAGATCTTGATGTTTGTGCTCGGGTTTTT | AATTAAAAACCCGAGCACAAACATCAAGATCTCGAGATCTTGATGTTTGTGCTCGGG |
| pcDNA-FOXK2 | GTACCGAGCTCGGATCCATGGCGGCGGCCGCGGCGGCGCTCTC | GGATATCTGCAGAATTCCTAGTTCTGGACACCCTTTTCC |
