# Supplementary material for: FOXK2 regulates fatty acid metabolism and promotes cervical cancer progression by activating the mTOR/DRP1 signaling axis
Source: Front Cell Dev Biol. 2025 Jun 26;13:1615454. doi: 10.3389/fcell.2025.1615454 (PMC12240978; doi:10.3389/fcell.2025.1615454)
Supplement: Supplementary file 2 [file Table2.docx]

**Table S2.** Primers for qRT-PCR.

| Gene | Forward | Reverse |
| --- | --- | --- |
| FOXK2 | AAGAACGGGGTATTCGTGGAC | CTCGGGAACCTGAATGTGC |
| ACC1 | TCACACCTGAAGACCTTAAAGCC | AGCCCACACTGCTTGTACTG |
| CPT1A | TCCAGTTGGCTTATCGTGGTG | TCCAGAGTCCGATTGATTTTTGC |
| FASN | AAGGACCTGTCTAGGTTTGATGC | TGGCTTCATAGGTGACTTCCA |
| GAPDH | GGAGCGAGATCCCTCCAAAAT | GGCTGTTGTCATACTTCTCATGG |
